# Supplementary material for: Radiosensitizing Chemotherapy (Irinotecan) with Stereotactic Body Radiation Therapy for the Treatment of Inoperable Liver and/or Lung Metastases of Colorectal Cancer
Source: Cancers (Basel). 2021 Jan 11;13(2):248. doi: 10.3390/cancers13020248 (PMC7827408; doi:10.3390/cancers13020248)
Supplement: Supplementary file 1 [file cancers-13-00248-s001.pdf]

Supplementary Materials

Radiosensitizing Chemotherapy (Irinotecan) with Stereotactic Body Radiation Therapy for the Treatment of Inoperable Liver and/or Lung Metastases of Colorectal Cancer

Radiotherapy protocol details

Treatment had to start three weeks maximum after inclusion. Two planning CT minimum (at least one contrast-enhanced for the liver) were required for the delineation of the CTV. An internal target volume (ITV) was defined depending on the techniques of SBRT (Body frame, Novalis, Brain Lab, Feldkirchen, Germany or Cyberknife, Accuray, Sunnyvalley, CA). The planning target volume (PTV) was defined as ITV + 3mm minimum laterally and in the antero-posterior direction and ITV+5mm minimum in the craniocaudal direction. Repositioning was achieved by stereotactic extracranial body framework (Body Frame Elekta) with cone-beam computed tomography (CBCT) or based on the detection of fiducials that were implanted close to the target. An intensity-modulated planning system was possible depending on the proximity of the target to the organs at risk (OAR).

Table 1 : Patient and treatment characteristics for the patient lost during the follow-up. Synchronous = time delay with primitive diagnosis ≤ 4 months.

		Lung
**Lost to follow-up patient characteristics**	**(1 patient)**	
**Age (years)**		62.2
**Performans Status**		Unknown
**Synchronous metastases with primitive diagnosis**		No
**Prior chemotherapy**		
	Adjuvant	Yes
	Metastatic lines	2
	Prior irinotecan	No
**Prior metastase-directed surgery**		
	Liver	No
	Lung	No
**Time between primitive diagnosis and inclusion (months)**		42.7
**Largest diameter (mm)**		
	Target 1	5
	Target 2	6
**Treatment characteristics**		
**Number of lesions**		2
	PTV volume (cm^3) (Target 1 – Target 2)	8.3-10.3
	Surfacic isodose 4x10 Gy	Yes
	Fiducials	No
	Respiratory monitoring	Yes

Table 2 : QLQ-C30 score differences (minimum ; first quartile ; median ; third quartile ; maximum) between baseline and 9-month. Mean values ± standard deviation are given at inclusion and 9-month.

Variable QLQ-C30 (13 patients)	Inclusion	9-month	Difference	P-value
Physical functioning	87.7±9.3	87.7±15.8	(-26.6; -6.6; 0; 6.6; 26.6)	1.00
Role functioning	84.6 ±17.3	88.4±28.3	(-50; -16.6; 0; 0; 66.6)	0.37
Emotional functioning	82.6±15.2	86.1±23.4	(-33.3; -25; 0; 4.16; 41.6)	0.54
Cognitive functioning	84.7±19.4	80.5±30	(-16.6; 0; 0; 4.16; 33.3)	0.50
Social functionning	86.1±22.3	81.9±32.1	(-33.3; -4.16; 0; 4.16; 66.6)	0.84
Global health status	75±16.6	76.4±26	(-25; -16.6; 0; 2.08; 50)	0.56
Fatigue	21.3±16.6	25.2±27.8	(-33.3; -22.2; 0; 0; 33.3)	0.42
Nausea/vomiting	1.28±4.6	3.8±9.9	(-16.6; 0; 0; 0; 0)	0.50
Pain	28.2±30.7	14.1±28.7	(-33.3; 0; 0; 33.33; 66.6)	0.06
Dyspnea	5.12±12.5	10.2±21	(-66.6; 0; 0; 0; 0)	1.00
Insomnia	20.5±32	15.4±22	(-33.3; 0; 0; 0; 100)	0.87
Appetite loss	0±0	5.12±12.5	(-33.3; 0; 0; 0; 0)	0.50
Constipation	11.11±16.4	13.9±17.1	(-33.3; 0; 0; 0; 33.3)	1.00
Diarrhea	5.5±12.9	5.5±12.9	(-33.3; 0; 0; 0; 33.3)	1.00
Financial difficulties	5.5±12.9	11.1±21.7	(-66.6; 0; 0; 0; 0)	1.00
